# Assessment of Proteinuria in Patients with Chronic Kidney Disease Stage 3: Albuminuria and Non-Albumin Proteinuria

**DOI:** 10.1371/journal.pone.0098261

**Published:** 2014-05-27

**Authors:** Simon D. S. Fraser, Paul J. Roderick, Natasha J. McIntyre, Scott Harris, Christopher McIntyre, Richard Fluck, Maarten W. Taal

**Affiliations:** 1 Academic Unit of Primary Care and Population Sciences, University of Southampton, Southampton, Hampshire, United Kingdom; 2 The Department of Renal Medicine, Royal Derby Hospital NHS Foundation Trust, Derby, Derbyshire, United Kingdom; 3 Division of Medical Sciences and Graduate-Entry Medicine, University of Nottingham, Nottingham, Nottinghamshire, United Kingdom; Mario Negri Institute for Pharmacological Research and Azienda Ospedaliera Ospedali Riuniti di Bergamo, Italy

## Abstract

**Background and Objective:**

Proteinuria assessment is key in investigating chronic kidney disease (CKD) but uncertainty exists regarding optimal methods. Albuminuria, reflecting glomerular damage, is usually measured, but non-albumin proteinuria (NAP), reflecting tubular damage, may be important. This study investigated the prevalence and associations of albuminuria and NAP, and the optimum number of urine specimens required.

**Methods:**

1,741 patients with CKD stage 3, recruited from primary care, underwent medical history, clinical assessment, blood sampling, and submitted three early morning urine samples for albumin to creatinine ratio (uACR) and protein to creatinine ratios (uPCR). Albuminuria was defined as uACR ≥3 mg/mmol in at least two of three samples. Isolated NAP was defined as uPCR ≥17 mg/mmol in two of three samples and uACR <3 mg/mmol in all three. Prevalence and associations of albuminuria and NAP, degree of agreement between single uACR and average of three uACRs, and urine albumin to protein ratio (uAPR = uACR/uPCR) were identified.

**Results:**

Albuminuria prevalence was 16% and NAP 6%. Using a <1 mg/mmol threshold for uACR reduced NAP prevalence to 3.6%. Independent associations of albuminuria were: males (OR 3.06 (95% CI, 2.23–4.19)), diabetes (OR 2.14 (1.53–3.00)), lower estimated glomerular filtration rate ((OR 2.06 (1.48–2.85) 30–44 vs 45–59), and high sensitivity CRP ((OR 1.70 (1.25–2.32)). NAP was independently associated with females (OR 6.79 (3.48–13.26)), age (OR 1.62 (1.02–2.56) 80 s vs 70–79) and high sensitivity CRP ((OR 1.74 (1.14–2.66)). Of those with uPCR≥17 mg/mmol, 62% had uAPR<0.4. Sensitivity of single uACR was 95%, specificity 98%, PPV 90%. Bland Altman plot one vs average of three uACRs showed: mean difference 0.0064 mg/mmol (SD 4.69, limits of agreement −9.19 to +9.20, absolute mean difference 0.837).

**Conclusions:**

In CKD stage 3, albuminuria has associations distinct from those of isolated NAP (except for inflammatory markers). Single uACR categorised albuminuria but average of three performed better for quantification.

## Introduction

The assessment of proteinuria is a key element of the investigation of kidney disease but some uncertainty exists regarding the optimal methods to apply. Specific unresolved issues include whether to measure total urinary protein and/or albuminuria and the optimum number of urine specimens required. People with chronic kidney disease (CKD) are at risk of mortality, cardiovascular disease (CVD) and less commonly progression to end stage renal disease (ESRD).[1], [2] Proteinuria, most often assessed as albuminuria, is a strong independent predictor of renal, cardiovascular, and mortality risk. [1], [3] An increasing level of urinary albumin to creatinine ratio (uACR) is independently associated with higher cardiovascular mortality risk and CKD progression. This association exists in both men and women, increases with age, and occurs in people with and without diabetes.[4]–[10] A single uACR measure has been used to derive risk in most cohort studies.[6]–[9]

Several CKD management guidelines, including those from the UK National Institute for Health and Clinical Excellence (NICE), the Kidney Disease Improving Global Outcomes (KDIGO) and the Kidney Disease Outcomes Quality Initiative (K/DOQI), recommend identification and quantification of proteinuria using uACR in preference to protein to creatinine ratio (uPCR).[11]–[13] In addition, some guidelines recommend repeating uACR measurements for initial identification of albuminuria to avoid over diagnosis due to transient albuminuria changes. [11], [14] It has been argued that uPCR is a more sensitive screening test for proteinuria; though uPCR and uACR perform similarly well in predicting adverse outcomes. [15], [16] Conversely, it could be argued that assessment of both albuminuria and non-albumin proteinuria (NAP) may provide valuable diagnostic and prognostic information. Albuminuria typically reflects glomerular disease, whereas NAP (including α2- and β2-microglobulins) is associated with tubulointerstitial pathology, and a low urinary albumin to total urinary protein ratio (uAPR) demonstrates strong correlation with tubulointerstitial disease on renal biopsy. [16]–[18] Some patients have a mixed proteinuria picture reflecting both glomerular and tubular dysfunction, particularly as total protein increases.[17] Little is known about the relative distributions of albuminuria and NAP in people with CKD, or the demographic and clinical associations of NAP or its prognostic significance.

This study aimed to investigate proteinuria assessment in a population of people with CKD stage 3 in a primary care setting in the UK, by determining the prevalence and associations of albuminuria and NAP, and assessing degree of agreement between a single uACR measure and two of three measures to identify albuminuria.

## Materials and Methods

The study was approved by Nottingham Research Ethics Committee 1. All participants provided written informed consent. The study was included on the National Institute for Health Research (NIHR) Clinical Research Portfolio (NIHR Study ID: 6632) and was independently audited by QED Clinical Services in November 2009. Participants were recruited for the Renal Risk in Derby (RRID) study, a prospective cohort of people with CKD stage 3 in a primary care setting. The methods for the RRID study have been published in detail elsewhere. [19] In summary, eligible participants were 18 years or over, met the Kidney Disease Outcomes Quality Initiative criteria for CKD stage 3 (estimated GFR (eGFR) between 30 to 59 ml/min per 1.73 m^2^ on two or more occasions at least 3 months apart prior to recruitment), were able to give informed consent, and were able to attend their general practitioner (GP) surgery for assessments. People who had previously had a solid organ transplant or who were terminally ill (expected survival <1 years) were excluded. The RRID study is conducted by a single nephrology department, but participants were recruited directly from 32 GP surgeries. Eligible patients were invited to participate via a letter sent by their GP and telephoned the coordinating centre to schedule a study visit. Study visits were conducted at participating GP surgeries by the researchers.

First study visits were conducted from August 2008 to March 2010. Screening and baseline visits were combined due to the large proportion of elderly participants and the logistical challenges associated with conducting study visits in multiple primary care centres. Participants were sent a medical and dietary questionnaire and three urine specimen bottles, and were asked not to eat cooked meat for at least 12 hours before the assessment. Urine was collected as three early morning samples on consecutive days before the study visit and was stored in a refrigerator. Specimens were submitted for analysis on the study visit day. Socioeconomic status was defined by the Indices of Multiple Deprivation score (IMD, a social deprivation score comprising a seven domain composite measure that demonstrates a strong relationship to health) and self-reported education status; an important indicator of socioeconomic status in elderly populations. [20], [21] Education status was grouped into three for analysis (group one: no formal qualifications, group two: General certificate of Secondary Education (GCSE) or equivalent, Advanced level (A level), or National Vocational Qualification (NVQ) 1–3, group three: first or higher degree, NVQ 4–5). Self-reported ethnicity information was collected. Due to small numbers of non-white participants, it was categorized into ‘White’ and ‘Other’ for the purposes of analysis.

At the assessment, information on questionnaires was checked, anthropomorphic measurements taken, and dipstick urinalysis performed. If this suggested a urinary tract infection, a specimen was submitted for microscopy and culture. Confirmed UTIs were treated with antibiotics and urine biochemistry was repeated after treatment. Blood specimens were taken and submitted for biochemical analysis (including serum creatinine, blood lipids and high sensitivity C–reactive protein (hsCRP, Roche Diagnostics, Newhaven, UK)). Creatinine was measured by a single autoanalyser using the Jaffe method and the assay was standardised against an isotope dilution mass spectrometry (IDMS) method. eGFR was calculated using the modified 4-variable Modified Diet in Renal Disease equation and categorised into four groups (>60, 45–59 (stage 3a), 30–44 (stage 3b), <30 (stages 4 and 5)). [22] Urine specimens were assayed for total protein, albumin and creatinine. Urine protein to creatinine ratio (uPCR) and uACR were calculated as measures of proteinuria. In defining proteinuria we chose the most conservative reported normal value for urinary protein excretion of <150 mg/day. Thus proteinuria was defined as uPCR≥17 mg/mmol (150 mg/g) in at least two of three samples. [23]

Non-albumin proteinuria was calculated as the difference between uPCR and uACR. Albuminuria was defined as KDIGO A2 or A3 (i.e. uACR≥3 mg/mmol) in at least two of three samples. For sensitivity analyses, those with ‘high normal’ albuminuria (uACR 1–3 mg/mmol) in at least two of three samples were also identified. Isolated NAP was defined as uPCR≥17 mg/mmol in two of three specimens and uACR<3 mg/mmol in all three specimens. For sensitivity analyses, we also defined isolated NAP as those with uPCR≥17 mg/mmol in two of three specimens and uACR<1 mg/mmol in order to exclude people with ‘high normal’ uACR from the NAP definition.

The urine albumin to protein ratio (uAPR) was calculated as the ratio of average of three uACRs divided by average of three uPCRs, and uAPR<0.4 was used as a cut off identified as having high sensitivity and specificity for primary tubulointerstitial disorders. [16]

BMI was calculated from weight in kilograms divided by height squared in metres and categorised according to WHO categories underweight (<18.5 kg/m^2^), normal (18.5–<25 kg/m^2^), overweight (25-<30 kg/m^2^), and obese (> = 30 kg/m^2^). [24] Central obesity was defined as a waist to hip ratio of ≥0.9 for men or ≥0.8 for women. [25] Diabetes was defined by having a previous clinical diagnosis in line with World Health Organization criteria. [26] Previous cardiovascular event was defined as subject-reported myocardial infarction, stroke, transient ischemic attack, revascularization, or amputation due to peripheral vascular disease, or aortic aneurysm. Smoking status was categorized as never smoked, ex-smoker, and current smoker. Taking a renin-angiotensin aldosterone system (RAAS) inhibitor was defined by chronic treatment with any dose of angiotensin converting enzyme inhibitor or angiotensin receptor blocker.

Blood pressure was measured after a minimum of five minutes rest in the sitting position, using a validated oscillometric device, recommended by the British Hypertension Society. The same device was used for all readings. BP was calculated as the mean of three readings that differed by <10%. Participants were asked ‘Were you told that you may have an issue with your kidneys before you were contacted to take part in this study?’ Those answering ‘yes’ were defined as being aware of their CKD diagnosis.

### Statistical analyses

Standard descriptive statistics were used to compare the characteristics of people with and without albuminuria and NAP. Univariate and multivariate logistic regression was used to identify the associations of albuminuria and isolated NAP, and of both albuminuria and NAP combined (rather than isolated albuminuria or isolated NAP). Interactions were assessed for age by gender to test for effect modification in age gender subgroups. Sensitivity analyses were conducted to assess the associations of isolated NAP excluding ‘high normal’ uACR. Two methods were used to compare a single measurement of uACR (from the first urine specimen) with three measures of uACR from the three specimens collected in this study. Firstly, the Bland Altman method was used to examine the degree of agreement between a single uACR measure and average of three uACR measures, considering albuminuria as a continuous variable in order to derive the mean difference (uACR-average uACR), mean absolute difference (i.e. ignoring the direction of any difference) and 95% limits of agreement (the values between which 95% of the differences would be expected to lie). [27] Secondly, considering albuminuria as a categorical variable, comparing a single uACR measure with having at least two of three uACR measures ≥3 mg/mmol.

All odds ratios are presented with 95% confidence intervals (CIs) and p values<0.05 are considered statistically significant. IBM SPSS Statistics for Windows version 19 was used to analyse the data.

## Results

### Whole group data

22% (1741) of approximately 8280 eligible participants from 32 GP practices invited to be included in the study agreed to participate (range 8–34% in different GP practices) and attended baseline assessment. 1052 (60%) were female, 1168 (67%) were over 70 years, and mean eGFR was 52.5 mL/min/1.73 m^2^ (SD 10.4). Median uACR was 0.33 mg/mmol, interquartile range 1.50. Median uPCR was 9.4 mg/mmol, interquartile range 7.63.Characteristics of people in the study are shown in table 1.

**Table 1:** Characteristics of people in the Renal Risk in Derby cohort.

Characteristic	Category	Total n = 1741 (numbers are n (% of total) unless otherwise stated)
**Gender**	Male	689 (39.6%)
	Female	1052 (60.4%)
**Age**	<60	128 (7.4%)
	60–69	445 (25.6%)
	70–79	761 (43.7%)
	80+	407 (23.4%)
**Ethnicity**	White	1698 (97.5%)
	Other	43 (2.5%)
**Index of multiple deprivation**	Quintile 1 (most deprived)	151 (8.7%)
	Quintile 2	432 (24.8%)
	Quintile 3	326 (18.7%)
	Quintile 4	447 (25.7%)
	Quintile 5 (least deprived)	382 (21.9%)
**Education status**	Group1 (No formal qualifications)	953 (54.7%)
	Group 2 (GCSE, Alevel, NVQ1-3)	469 (26.9%)
	Group 3 (1st or higher degree, NVQ4–5)	317 (18.2%)
**eGFR at study entry**	Mean (SD)	49.88 (8.08)
	>60	418 (24.0%)
	45–59	911 (52.3%)
	30–44	386 (22.2%)
	<30	26 (1.5)
**Proteinuria - based on 2 of 3 samples**	uPCR≥17 mg/mmol (150 mg/g)	298 (17.1%)
**Albuminuria – based on single sample**	No albuminuria	1440 (82.7%)
	Albuminuria A2 (≥3 mg/mmol but <30 mg/mmol)	247 (14.2%)
	Albuminuria A3 (≥30 mg/mmol)	44 (2.5%)
**Albuminuria - based on 2 of 3 samples**	No albuminuria	1470 (84.4%)
	Albuminuria A2 ≥3 mg/mmol but <30 mg/mmol)	231 (13.3%)
	Albuminuria A3 (≥30 mg/mmol)	40(2.3%)
**Non-albumin proteinuria**	uPCR≥17 mg/mmol (in at least 2 of 3 samples) and uACR<3 mg/mmol (in all samples)	105 (6.0%)
**Diabetes**	Yes	294 (16.9%)
	No	1447 (83.1%)
**Hypertension**	On antihypertensive medication	1426 (81.9%)
	BP>140/90 at study assessment, but not on antihypertensive medication	102 (5.9%)
	No hypertension	213 (12.2%)
**Number of antihypertensive medications**	None	315 (18.1%)
	1	615 (35.3%)
	2	488 (28.0%)
	3 or more	323 (18.6%)
**Taking RAASi**	Yes	1123 (64.5%)
	No	618 (35.5%)
**History of CVD**	Yes	592 (34.0%)
	No	1149 (66.0%)
**Smoking**	Current	81 (4.7%)
	Ex-smoker	866 (49.7%)
	Never	794 (45.6%)
**Alcohol**	No alcohol	711 (40.8%)
	Drinking within recommended limits	877 (50.4%)
	Drinking above recommended limits	65 (3.7%)
**BMI**	Normal or underweight	353 (20.3%)
	Overweight	738 (42.4%)
	Obese	650 (37.3%)
**Central obesity**	Yes	1480 (85.0%)
	No	260 (14.9%)
**Total cholesterol to HDL ratio**	>4.5	306 (17.6%)
**High sensitivity CRP**	Median (inter-quartile range)	2.23 (1.14–4.59)
**Aware of CKD diagnosis**	Yes	1026 (58.9%)
	No	715 (41.1%)

### Prevalence of proteinuria

**Figure 1:**
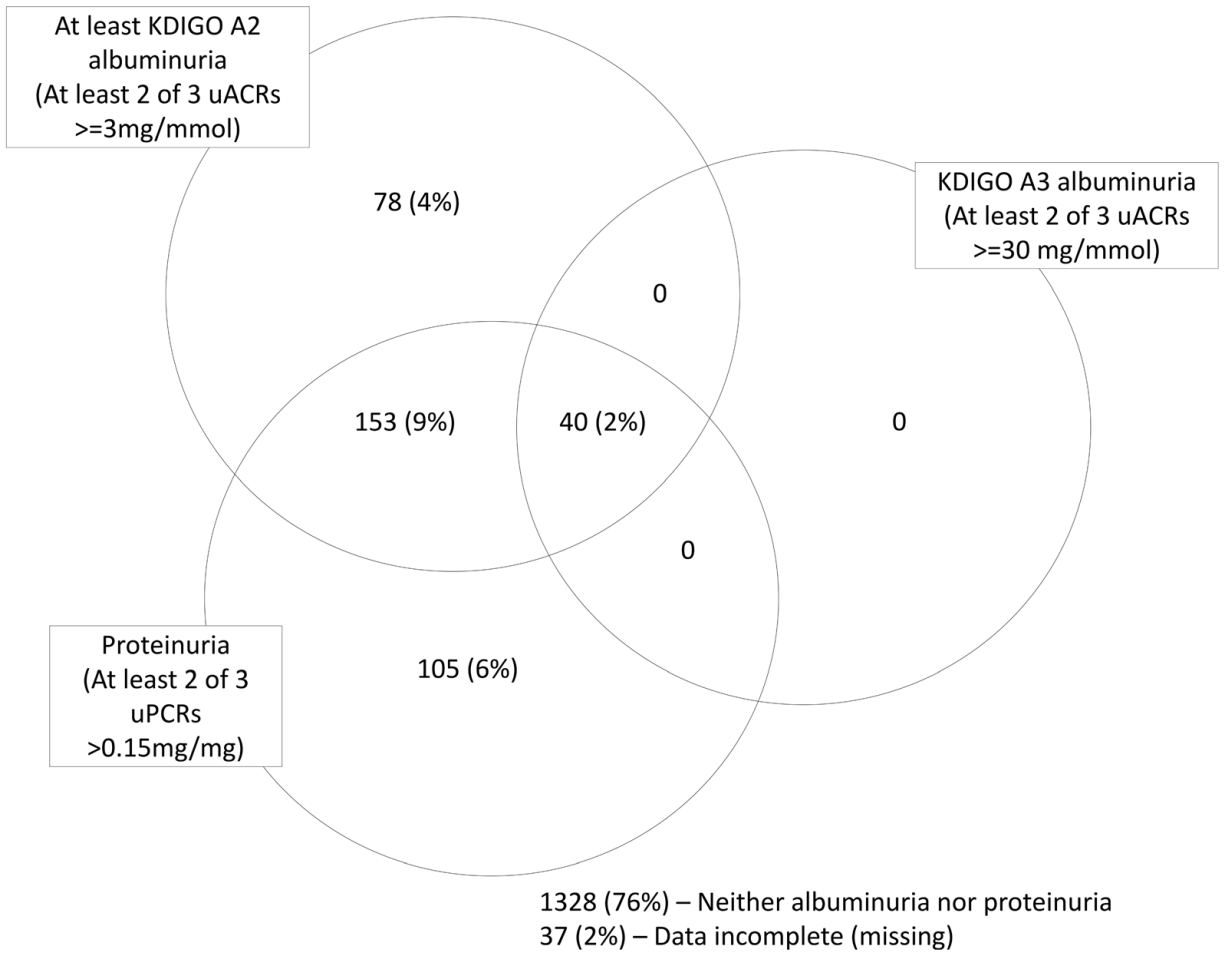
The distribution of albuminuria (based on 2 of 3 uACRs) and non-albumin proteinuria (based on 2 of 3 uPCRs) in people with chronic kidney disease stage 3 in the Renal Risk in Derby study.

**Figure 2:**
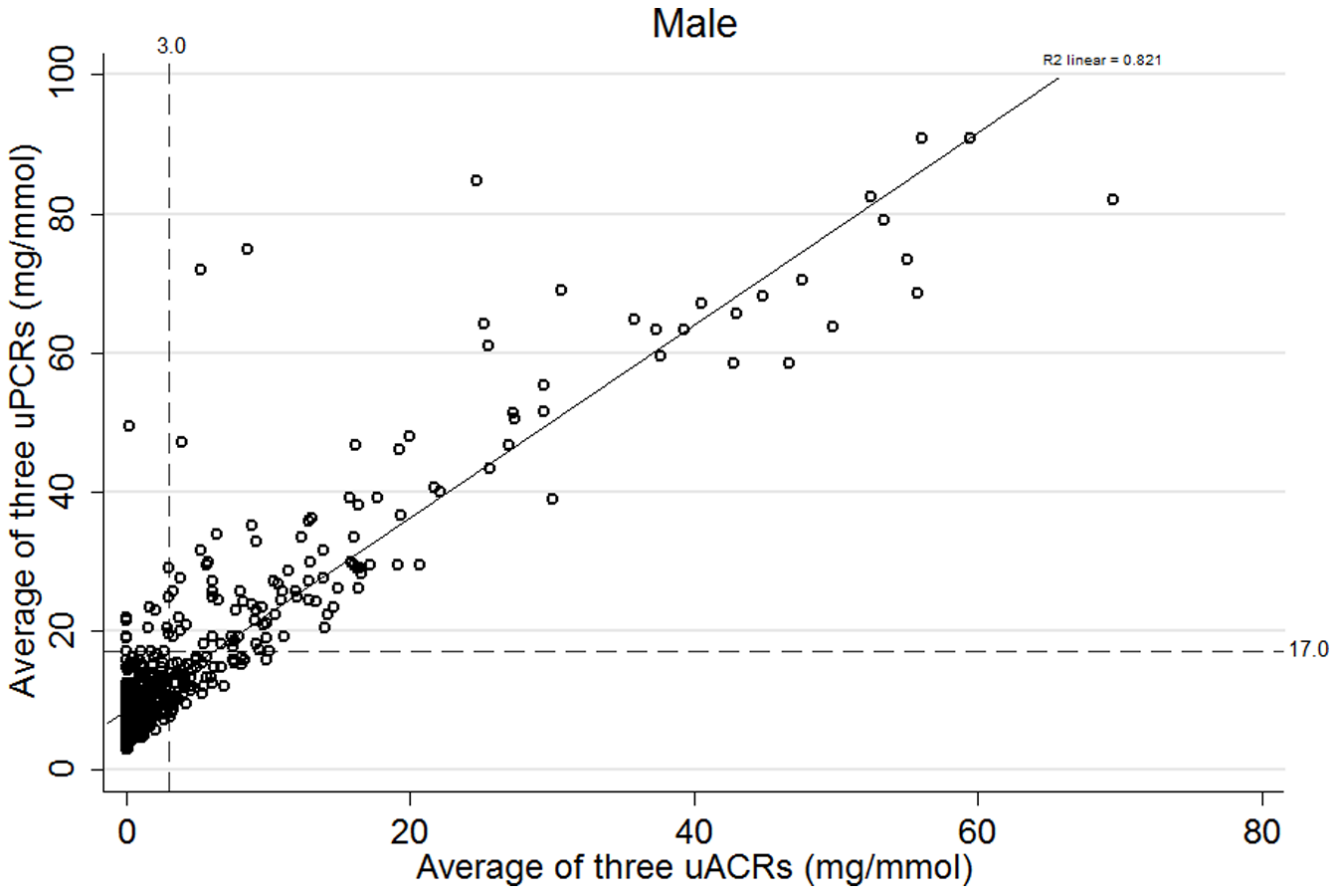
Scatterplot showing the distribution of uACR and uPCR relative to clinically important threshold values for males (excluding outlier values (ACR over 70 mg/mmol and PCR 150 mg/mmol)). Explanatory footnote for Figure 2: High values have been excluded (uACR>70 mg/mmol and uPCR>150 mg/mmol) to better illustrate the relationship at lower levels of proteinuria.

**Figure 3:**
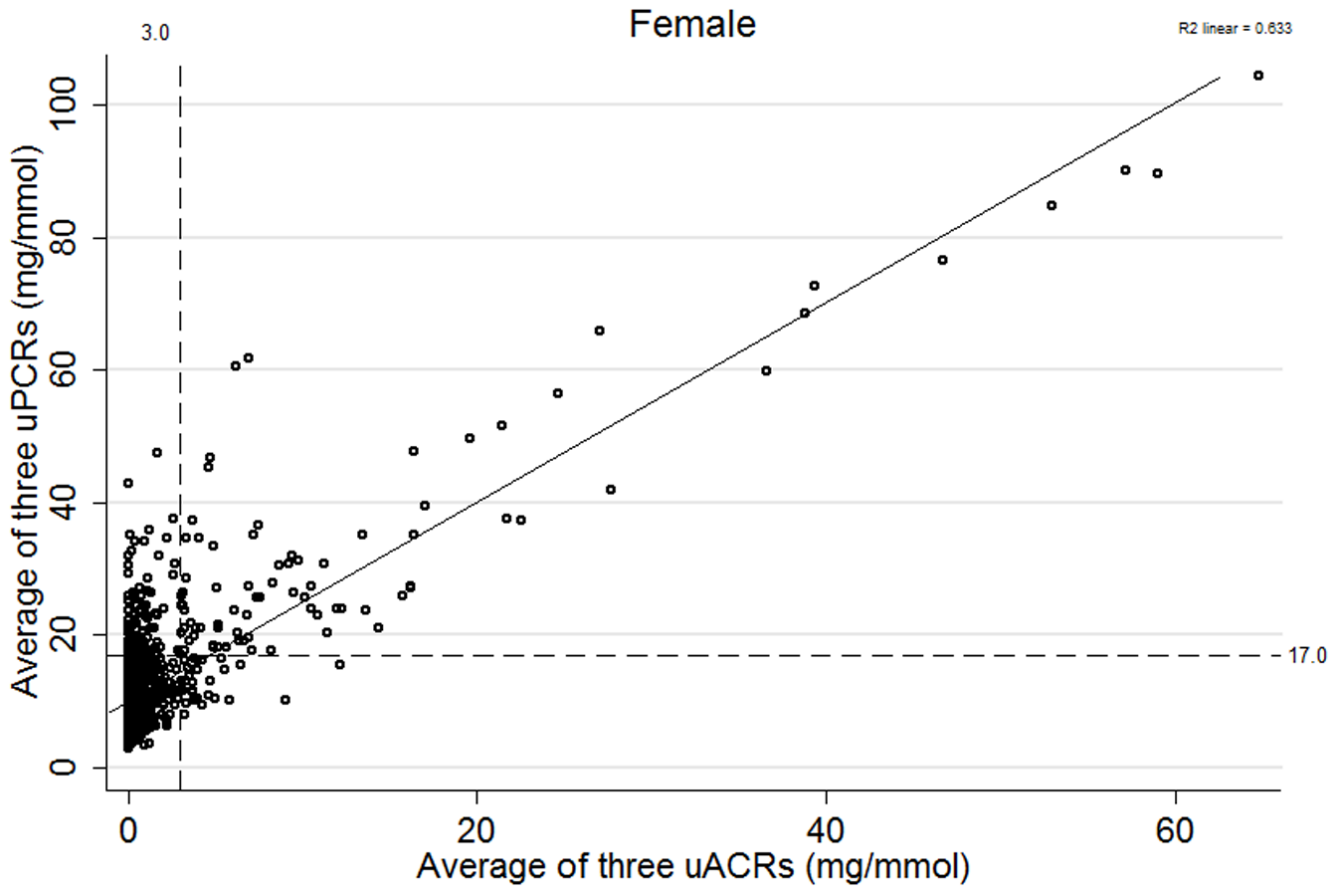
Scatterplot showing the distribution of uACR and uPCR relative to clinically important threshold values for females (excluding outlier values (ACR over 70 mg/mmol and PCR 150 mg/mmol)). Explanatory footnote for Figure 3: High values have been excluded (uACR>70 mg/mmol and uPCR>150 mg/mmol) to better illustrate the relationship at lower levels of proteinuria.

Proteinuria of any type (albuminuria (KDIGO A2 or A3) and isolated NAP) was present in 376 people (22%) (Figure 1). 271 people (16%) had albuminuria based on two of three uACR positive measures whereas 291 (17%) had abnormal uACR on first single uACR. Of those with confirmed albuminuria, 78 (4%) had isolated albuminuria and 193 (11%) had mixed albuminuria and NAP. 105 people (6%) had isolated NAP (on two of three uPCRs). 539 people (31%) had ‘high normal’ albuminuria (uACR 1–3 mg/mmol). Defining isolated NAP using a <1 mg/mmol threshold for uACR reduced the isolated NAP prevalence to 63 (3.6%). The distribution of uACR and uPCR for males and females is shown in Figures 2 and 3 respectively with threshold values. These plots demonstrate that there are significant numbers of people (particularly women) with isolated NAP as they fall below the threshold for albuminuria. There are also some (more noticeable in men) who have albuminuria but fall below the threshold for proteinuria.

### Albumin to protein ratio

Of those with uPCR≥17 mg/mmol (n = 298), 185 (62%) had uAPR<0.4 suggesting predominantly tubulointerstitial pathology; 45 (24%) were male and 140 (76%) were female.

### Associations with proteinuria

Univariate associations with albuminuria were male gender, low eGFR, diabetes, hypertension, smoking, history of CVD, raised cholesterol/HDL ratio, lower educational attainment and hsCRP. In multivariate analysis, the only significant positive associations of albuminuria were with male gender, lower eGFR, diabetes and hsCRP (Table 2). There was no association with SES measured by IMD or education status in the fully adjusted model. Defining albuminuria by single uACR measure or two of three uACRs did not alter these associations.

**Table 2:** Associations of having at least A2 albuminuria in at least two of the three urine samples in the Renal Risk in Derby cohort (n with 3 uACR results = 1736).

		Univariate		Multivariate †		Multivariate ††	
		OR (95%CI)	p	OR (95%CI)	p	OR (95%CI)	p
Gender (compared to female)	Male	3.42 (2.61–4.48)	<0.001	2.96 (2.17–4.03)	<0.001	3.06 (2.23–4.19)	<0.001
Age (as continuous)		1.01 (1.00–1.02)	0.20	0.99 (0.97–1.00)	0.08	0.99 (0.97–1.00)	0.10
Deprivation (compared to Quintile 5, least deprived)	Quintile 1 (most deprived)	0.98 (0.57–1.69)	0.18*	-	-	-	-
	Quintile 2	1.10 (0.74–1.62)		-	-	-	-
	Quintile 3	0.97 (0.64–1.49)		-	-	-	-
	Quintile 4	1.45 (1.00–2.10)		-	-	-	-
Education (compared to Group 1, people with no formal qualifications)	Group 2 (GCSE,Alevel,NVQ1-3)	0.72 (0.52–0.99)	0.02*	0.73 (0.50-1.07)	0.18*	-	-
	Group 3 (1st or higher degree,NVQ4-5)	0.58 (0.39–0.85)		0.69 (0.45-1.06)		-	-
eGFR at study entry (compared to eGFR 45–59)	<30	4.65 (2.09–10.37)	<0.001*	3.24 (1.27–8.28)	<0.001*	3.13 (1.21–8.06)	<0.001*
	30–44	2.59 (1.94–3.47)		2.17 (1.57–3.00)		2.06 (1.48–2.85)	
	>60	0.40 (0.26–0.63)		0.42 (0.25–0.69)		0.47 (0.29–0.79)	
Diabetes (compared to people without diabetes)	People with diabetes	2.68 (1.99–3.60)	<0.001	-	-	2.14 (1.53–3.00)	<0.001
Hypertension (compared to people without hypertension)	People with hypertension	3.14 (1.76–5.59)	<0.001	-	-	1.80 (0.95–3.40)	0.07
History of CVD (compared to people without CVD)	People with CVD history	1.44 (1.11–1.88)	<0.01	-	-	-	-
Smoking (compared to never smokers)	Current smokers	2.88 (1.70–4.89)	<0.001*	-	-	1.80 (0.95–3.40)	0.19*
	Ex-smokers	1.56 (1.18–2.06)		-	-	1.04 (0.75–1.44)	
BMI (compared to normal BMI)	Overweight	0.90 (0.63–1.26)	0.44*	-	-	-	-
	Obese	0.86 (0.60–1.22)		-	-	-	-
Central obesity (compared to people not centrally obese)	Central obesity	0.96 (0.67–1.37)	0.80	-	-	-	-
Elevated lipid ratio (compared to people with normal ratio)	Total cholesterol/HDL>4.5	1.38 (1.00–1.89)	0.05	-	-	-	-
High sensitivity CRP (log transformed, as continuous)		1.88 (1.41–2.51)	<0.001	1.74 (1.28–2.37)	<0.001	1.70 (1.25–2.32)	0.001

† Model adjusted for age, sex, education, eGFR, HsCRP.

†† Model adjusted for age, sex, diabetes, hypertension, smoking, eGFR, HsCRP.

*p value shown for variables with more than one group is p for trend.

By contrast, isolated NAP was strongly positively associated with female gender and increasing age, and also associated with elevated cholesterol/HDL ratio, hsCRP, being aware of CKD diagnosis and lower SES (defined by education status). The associations with female gender, age and hsCRP remained in the multivariable model. No interactions were observed (Table 3). Sensitivity analyses using isolated NAP defined by a <1 mg/mmol threshold for uACR (to exclude ‘high normal’ uACR) did not alter these associations.

**Table 3:** Associations of having isolated non-albumin proteinuria in the Renal Risk in Derby cohort (based on two of three uACRs and two of three uPCRs).

		Univariate		Multivariate †		Multivariate ††	
		OR (95%CI)	p	OR (95%CI)	p	OR (95%CI)	P
Gender (compared to male)	Female	6.82 (3.53–13.18)	<0.001	7.49 (3.86–14.50)	<0.001	6.80 (3.47–13.30)	<0.001
Age (as continuous)		1.05 (1.02–1.07)	<0.001	1.06 (1.03–1.08)	<0.001	1.05 (1.02–1.07)	0.001
Deprivation (compared to Quintile 5, least deprived)	Quintile 1 (most deprived)	1.95 (0.95–4.00)	0.19*	-	-	-	-
	Quintile 2	1.53 (0.85–2.74)		-	-	-	-
	Quintile 3	1.25 (0.65–2.38)		-	-	-	-
	Quintile 4	0.94 (0.50–1.78)		-	-	-	-
Education (compared to Group 3 (1st or higher degree, NVQ4-5))	No formal qualifications	2.34 (1.23–4.47)	0.01*	-	-	1.25 (0.64–2.46)	0.33*
	Group 2 (GCSE, Alevel, NVQ1-3)	1.30 (0.62–2.74)		-	-	1.38 (0.92–2.09)	
eGFR (compared to 45-59)	<30	1.38 (0.32–5.98)	0.45*	-	-	-	-
	30–44	0.90 (0.53–1.53)		-	-	-	-
	>60	1.37 (0.87–2.16)		-	-	-	-
Knowledge of CKD (compared to people not aware of their CKD diagnosis)	People aware of their CKD diagnosis	1.59 (1.08–2.36)	0.02	-	-	1.38 (0.92–2.09)	0.12
Diabetes (compared to people without diabetes)	People with diabetes	1.16 (0.70–1.91)	0.58	-	-	-	-
Hypertension (compared to people without hypertension)	People with hypertension	1.00 (0.55–1.82)	0.99	-	-	-	-
History of CVD (compared to people without CVD)	People with CVD history	1.04 (0.69–1.58)	0.84	-	-	-	-
Smoking (compared to never smokers)	Current smokers	0.92 (0.36–2.37)	0.63*	-	-	-	-
	Ex-smokers	0.82 (0.55–1.23)		-	-	-	-
BMI (compared to normal BMI)	Overweight	0.86 (0.60–1.22)	0.44*	-	-	-	-
	Obese	0.90 (0.63–1.26)		-	-	-	-
Central obesity (compared to people not centrally obese)	Central obesity	1.07 (0.61–1.88)	0.81	-	-	-	-
Elevated lipid ratio (compared to people with normal ratio)	Total cholesterol/HDL >4.5	0.42 (0.21–0.83)	0.01	-	-	0.51 (0.25–1.03)	0.06
Taking RAASi (compared to those not taking	Taking RAASi	0.73 (0.49–1.09)	0.12	-	-		
High sensitivity CRP (log transformed, as continuous)		1.68 (1.12–2.52)	0.01	-	-	1.74 (1.14–2.66)	0.01

† Model adjusted for age and gender only.

†† Model adjusted for age, gender, education status, knowledge of CKD, cholesterol/HDL ratio and HsCRP.

*p value shown for variables with more than one group is p for trend.

Having both albuminuria and NAP (n = 193) was positively associated with male gender, diabetes, hypertension, smoking, being aware of CKD diagnosis and hsCRP on univariate analysis. The associations with male gender, diabetes, smoking, being aware of CKD diagnosis and hsCRP remained after full adjustment (data not shown).

### One versus multiple measures of proteinuria

**Figure 4:**
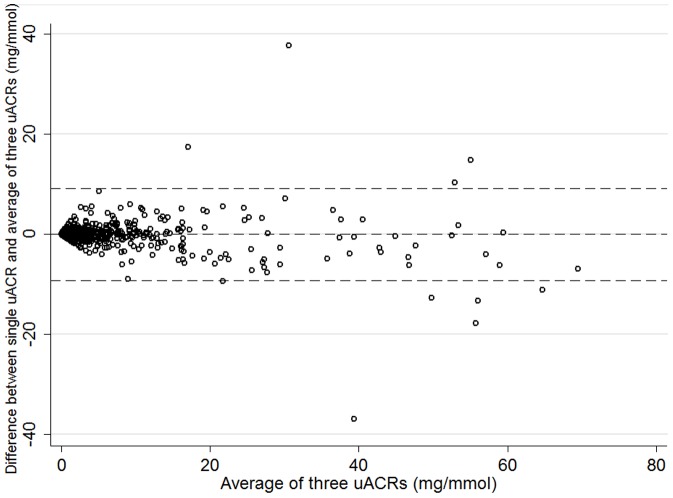
Bland Altman plot showing the distribution of the average of three uACRs against the difference between one uACR and the average of three (figure restricted to people with average uACR<70 mg/mmol). Explanatory footnote for Figure 4: Horizontal lines represent the mean difference and the 95% limits of agreement in the whole study population (upper = 9.20, lower = −9.19, mean = 0.0064)

Comparing one vs average of three measures of uACR, the mean difference was 0.0064 mg/mmol (uACR-average uACR = 0.0064, SD 4.69, 95% limits of agreement −9.19 to +9.20 mg/mmol) (Figure 4). In contrast, the mean absolute difference (i.e. ignoring the direction of any difference was 0.837 mg/mmol (SD4.62)). In quantifying albuminuria it is the absolute difference that is most relevant. Comparing single and multiple uACR measures as categorical variables there was disagreement between one measure of uACR and three for only 45/1734 (2.6%). Considering the presence albuminuria in at least 2 of 3 specimens as the reference test, the sensitivity of one uACR was 94.6%, specificity 97.9%, and positive predictive value 89.8%. Sensitivity of a single PCR (compared to two of three) for isolated NAP was 81%, specificity was 95%, and positive predictive value was 48% (prevalence of isolated NAP was only 6%).

## Discussion

In this cohort of 1741 people with CKD stage 3, albuminuria was present in 16% and was associated with NAP in 11%. Isolated NAP was present in 6%. Presence of albuminuria was associated with male gender, diabetes, lower eGFR and hsCRP, whereas the pattern of isolated NAP was different – it was associated with female gender, increasing age and hsCRP. A single uACR was sufficient for identification of albuminuria, but due to intra-individual variation in uACR three measurements are preferable for quantification of albuminuria.

Albuminuria was relatively uncommon in this cohort suggesting that most people with CKD stage 3 in primary care have tubulo-interstitial and/or vascular rather than glomerular pathology. This low prevalence of albuminuria and the strong association with adverse renal and cardiovascular outcomes underlies the importance of detection to identify the minority of people with CKD who are at increased risk. [5], [28], [29]

We identified a small number of people with isolated NAP who would not have been identified by use of uACR alone and demonstrated clear differences in albuminuria and NAP distribution patterns in people with CKD 3. We also identified a sub-group of people with both albumin and non-albumin proteinuria. A sensitivity analysis showed that NAP was also associated with “high normal” albuminuria in 40% of cases. Nevertheless, these observations are consistent with albuminuria and NAP reflecting different renal pathologies (glomerular and tubulo-interstitial). The associations we observed with albuminuria were similar to those reported from the CRIC study, except for lack of association with BMI in our study.[30] The association between albuminuria and diabetes likely reflects diabetes being a common cause of glomerulopathy. In retrospective analyses of uACR and uPCR in people with CKD, Methven et al identified the high sensitivity of uPCR as a test to identify ‘clinically relevant’ proteinuria (compared with 24-hour urine collection), and stressed the equivalence of uACR and uPCR in predicting renal outcomes and mortality. [15], [16] In a separate study they reported increased risk of death (HR2.34 (95%CI 1.63–3.35)), renal replacement therapy (HR2.90 (95%CI 1.31–6.43)), and CKD progression (HR for doubled serum creatinine 2.35 (95% CI 1.62–3.40)) among people with discordant (i.e. predominantly non-albumin) proteinuria (low ACR(<30 mg/mmol), high PCR(≥50 mg/mmol)).[17] However the magnitude of proteinuria in this study was higher than our population and different thresholds were used. A study comparing presence of NAP (identified by a low APR) with histology from renal biopsy, confirmed the association with tubulointerstitial pathology. [18] While the prognostic significance of isolated NAP remains unknown, our findings suggest that identification of NAP may provide additional diagnostic information in certain groups of people, particularly older women. The low positive predictive value of a single uPCR for isolated NAP in this study suggests that future prognostic studies would benefit from using more than one measure. This is important in the light of the recent suggestion that at least a proportion of NAP may be artefactual.[31] It is of interest that a marker of inflammation (hsCRP) was associated with both albuminuria and NAP in this study. Systemic inflammation is well recognised to be associated with increased cardiovascular and progression risk in people with CKD.[32], [33] While the association with albuminuria is not surprising, the association with NAP was unexpected and future research into the prognostic significance of NAP should also consider the role of inflammation.

Use of a single urine specimen uACR to define albuminuria misclassified some individuals in this study compared to using two of three specimens, but sensitivity, specificity and positive predictive value of a single uACR were high. Our use of ‘two of three’ uACRs and uPCRs differs from many studies that have used a single uACR to identify albuminuria and determine prognosis.[5]–[9] The HUNT 2 study used the mean of three uACR values to define albuminuria, but did not make comparison with a single value.[4] Our more restricted definition of albuminuria aimed to improve specificity and better reflect clinical practice (in which albuminuria identified in primary care is confirmed by repeat testing).[11] However, our findings suggest that a single measure of uACR is sufficient to categorise albuminuria for clinical decision making. Conversely, we have confirmed substantial intra-individual variation in uACR values on consecutive days, suggesting that an average of three uACR measurements is preferable for quantification of albuminuria. The predictive model developed by Tangri et al. for progression of CKD to ESRD was based on a single ACR measure.[2] Our results suggest that further research on risk prediction models in CKD should also compare the use of more than one ACR value.

This study had several strengths, including large numbers of people with CKD, being conducted in a primary care setting, standardisation of measures, and the use of three morning urine samples to assess albuminuria and proteinuria. However, it has several limitations. These analyses were cross sectional, limiting the ability to infer causality. The majority of patients were already treated with RAASi at baseline, which may have masked proteinuria and therefore underestimated true proteinuria prevalence. The 2012 KDIGO clinical practice guidelines recommended three categories of albuminuria to grade risk – A1 normal to mildly increased (<3 mg/mmol), A2 moderately increased (3–30 mg/mmol), and A3 severely increased (>30 mg/mmol).[14] We chose to examine associations with A2 and A3 combined because the absolute numbers with A3 (ACR>30 mg/mmol) were small (41 people  = 2.4%) and increased risk has been demonstrated for all grades of albuminuria.[4] Non-response to recruitment could have caused selection bias, and the predominantly elderly population could result in survivor bias. This means that caution should be used applying these results to other CKD populations. However, our population was similar to larger studies in the UK primary care setting, suggesting that this was broadly representative of people with CKD in this context.[34] Further limitations were that we had no specific measures of tubular dysfunction or Bence Jones protein to relate to NAP, and that prevalence of NAP may have been increased by the high proportion of people taking RAAS inhibitors.

### Conclusions

Although albuminuria is relatively uncommon in CKD stage 3 in primary care, its identification is important to determine prognosis and guide intervention. Albuminuria and isolated NAP have distinct associations in people with CKD stage 3, likely reflecting glomerular and tubulo-interstitial pathology respectively. The prognostic significance of isolated NAP needs further assessment. A single measure of uACR is sufficient to categorise albuminuria but three uACR measurements are preferable for quantification.
